# Mental health symptoms among the nurses of Bangladesh during the COVID-19 pandemic

**DOI:** 10.1186/s43045-021-00103-x

**Published:** 2021-04-02

**Authors:** Saifur Rahman Chowdhury, Tachlima Chowdhury Sunna, Dipak Chandra Das, Humayun Kabir, Ahmed Hossain, Sabbir Mahmud, Shakil Ahmed

**Affiliations:** 1 Department of Public Health, North South University, Dhaka, Bangladesh; 2 Global Health Institute, North South University, Dhaka, Bangladesh; 3 Department of Surgery, Bangabandhu Sheikh Mujib Medical University, Dhaka, Bangladesh

**Keywords:** Anxiety, Depression, Healthcare workers, Psychological impact, Stress

## Abstract

**Background:**

The coronavirus disease-19 (COVID-19) pandemic has wreaked havoc on healthcare staff and caused serious psychological distress. We aimed to determine the effects of the COVID-19 pandemic on the mental health of Bangladeshi nurses, as well as the relationship between occupational factors and mental health symptoms. We conducted a cross-sectional study among registered nurses in Bangladesh. We used the Depression, Anxiety and Stress Scale-21 (DASS-21) and the Impact of Event Scale-Revised (IES-R) to assess mental health symptoms.

**Results:**

Among the 547 nurses included in the study, the prevalence of mild to extremely severe depression, anxiety, and stress was 50.5%, 51.8%, and 41.7% respectively, and 61.9% of the respondents reported mild to severe psychological impact for COVID-19. Psychological symptoms were more prevalent among female nurses than male nurses (*p <0.05*). Linear regression revealed that having complete personal protective equipment (PPE) during working was significantly associated with lower levels of depression, anxiety, and stress (*p <0.05*). Facing any emotional abuse working in the COVID-19 pandemic situation was significantly associated with higher levels of depression, anxiety, and stress and greater psychological impact of the outbreak (*p <0.05*).

**Conclusions:**

A high prevalence of mental health symptoms was observed in nurses. We recommend the implementation of mental well-being interventions and ensuring a proper work environment for nurses during the pandemic.

## Background

From the beginning of 2020, coronavirus disease 2019 (COVID-19) has become an international concern [1]. This severe infectious disease was first detected in China at Wuhan City of Hubei province in December of 2019 [2]. Due to the rapid transmission of the disease, it was announced as an international public health emergency by the World Health Organization (WHO) on 30 January 2020 [3] and as a pandemic on 11 March 2020 [4]. The number of COVID-19 cases has risen exponentially worldwide. As of 31 March 2021, it has been documented that the number of people with COVID-19 is approximately 128 million, and the number of death is more than 2.8 million [5].

Bangladesh is also passing through a critical situation from the beginning of the COVID-19 outbreak on 8 March 2020. More than six hundred thousand cases have been diagnosed, and 9,046 have died of COVID-19 [6]. The infection rate has escalated exponentially since April 2020 though the government of Bangladesh has considered different preventive measures [7], and other health services have been augmented in the country [7, 8]. As COVID-19 is highly contagious, it has spread across the country in a brief period. Thus, it has brought challenges to the fragile health system of Bangladesh [7, 9]. The health workers have played a significant role in tackling the ongoing pandemic from the very beginning. Though the Bangladeshi government recruited around five thousand nurses in this COVID-19 pandemic [10], there has still been a 76% shortage of nurses where the ratio of nurses to every 10,000 population is about only 3 [11, 12]. However, while working and treating in the COVID-19 pandemic, healthcare workers have been infected by the disease, and among them, a significant number are nurses [13].

Health care workers working in this pandemic situation are facing extreme levels of stressful or traumatic events that result in adverse mental health and psychological outcomes [14]. A study conducted in Singapore revealed higher levels of adverse mental health status and psychological outcomes (e.g., anxiety, depression, stress, and PTSD) among the health professionals who were caring for patients with COVID-19 [15]. Besides, the nurses were at a greater risk of anxiety than doctors as they were directly exposed to the COVID-19-positive patients [16]. A previous study found that inadequacy in precautionary measures in the workplace affects the healthcare professionals’ mental health [17]. The shortage of proper personal protective equipment (PPE) supply among the healthcare professionals from the respective authorities of almost every hospital of Bangladesh regardless of public or private settings has increased the risk of getting infected, and it has created havoc among this professional group [18]. Besides, nurses deal with COVID-19 patients directly and frequently; they have to face social stigma and violence that might interact with adverse mental health outcomes, too [19].

No evidence-based data on nurses’ psychological symptoms amid the COVID-19 situation in Bangladesh is currently available. Therefore, it is crucial to know about the status of mental health and psychological outcomes of nurses working in the COVID-19 pandemic to smoothly tackle this pandemic and be prepared for any other emergency. This study aimed to explore the prevalence of depression, anxiety, stress, and impact of COVID-19 among the nurses. This study also aimed to identify the potential occupational factors associated with mental health outcomes among the nurses in Bangladesh.

## Methods

### Study design

It is a cross-sectional, web-based survey that collected data between 22 November and 6 December 2020.

### Study participants

Convenience sampling technique was used to collect data. Because of the COVID-19 outbreak, close contact was suggested to be minimized among people; potential participants were electronically invited within the existing study participants. A link to the survey questionnaire was sent to the potential respondents through posting on social media and text messages. The online survey was disseminated to 1,000 registered nurses of 20 private and eight government hospitals covering 18 districts (out of 64 districts) in Bangladesh. In Bangladesh, nurses appear at the licensing examination to become registered for practicing after passing their diploma or bachelor program. According to the Bangladesh Nursing and Midwifery Council (BNMC), there are 66,973 registered nurses in Bangladesh currently in service. Study participants were male and female registered nurses of any age (as long as they were in employment) and of any grade (e.g., senior, junior).

### Questionnaire development

The online questionnaire was produced in “Google Forms” and written in English. When the respondents clicked on the link, they were informed about the survey nature and purpose on the first page. Subsequently, when the respondents decided to participate, they were transferred to the next page of the questionnaire (first section). Socio-demographic data, including age, gender, area of residence, marital status, and occupational details, including type of healthcare facility, type of job, level of education, professional title, working position, had complete PPE during working, and faced any emotional abuse (for being healthcare workers and working in the COVID-19 pandemic situation), were collected in the first part of the survey questionnaire. The area of residence of the respondents was categorized as divisional city, district town, and sub-district. In Bangladesh, a division is the first-level administrative area, and the divisions are divided into districts, where the sub-district functions as the sub-unit of the district. The infrastructure of healthcare facilities of Bangladesh is divided into three levels: tertiary level (medical universities, medical college hospitals, and specialty hospitals), secondary level (district hospitals, maternal and child welfare centers), and primary level (upazila (sub-unit of district) health complex, union (sub-unit of upazila) health and family welfare centers, community clinics). Regarding the professional title, the nurses who perform technical works are referred to as senior staff nurses, and those who perform administrative works are referred to as nurse in-charge. To ascertain the working position of the nurses, participants were questioned if they were specifically engaged in treating patients with or suspected of having COVID-19 in COVID-dedicated hospitals. Those who answered yes were identified as nurses of the frontline, and those who answered no were identified as nurses of the second line.

Psychological well-being status, which was measured using the “Depression, Anxiety and Stress Scale-21 (DASS-21),” was the second component of the study. The last part of the questionnaire was formed using the “Impact of Event Scale-Revised (IES-R)” to measure the psychological impact of COVID-19 on the respondents.

### Measurement of mental health symptoms

The “DASS-21 (Depression, Anxiety and Stress Scale-21)” is a 21-item questionnaire, collection of three self-administered scales designed to assess depression, anxiety, and stress [20]. The total subscale score of depression was divided into five categories: “normal” (0–9), “mild depression” (10–13), “moderate depression” (14–20), “severe depression” (21–27), and “extremely severe depression” (28–42). The anxiety subscale score was separated into “normal” (0–7), “mild anxiety” (8–9), “moderate anxiety” (10–14), “severe anxiety” (15–19), and “extremely severe anxiety” (20–42). The total subscale score of stress was also distributed into five categories: “normal” (0–14), “mild stress” (15–18), “moderate stress” (19–25), “severe stress” (26–33), and “extremely severe stress” (34–42). The DASS-21 is a validated screening instrument for evaluating mental health status and was also used in previous surveys to ascertain the psychological status in the COVID-19 pandemic [21–23]. The “IES-R (Impact of Event Scale-Revised)” is a 22-item self-report scale [24], which was validated before for measuring the degree of psychological impact after exposure to a public health emergency and that has been used in previous studies to determine the impact of COVID-19 [21–23]. It consists of 3 subscales, which are intrusion, avoidance, and hyperarousal. Respondents were asked to rate the level of distress for each item of the questionnaire during the last 7 days of their interview. Categorization of the score ranges from 0 to 23 for normal, 24 to 32 for mild, 33 to 36 for moderate, and more than 36 for severe psychological impact respectively. A cut-off score of 24 was used to describe the impact of the event of clinical concern [24, 25].

### Statistical analysis

Descriptive statistics were performed for the demographic variables and occupational characteristics. The IES-R and the DASS-21 subscale scores were expressed as mean and standard deviation. The severity categories, which are derived from the counts of each level for distress of depression, anxiety, stress, and impact of event, are presented as numbers and percentages. The nonparametric chi-square test was applied to determine the difference of each distress between genders. A linear regression model was fitted to explore the univariate associations of the selected psychological outcomes with occupational factors. A *p*-value of <0.05 was considered as significant in this study. The data were analyzed by using SPSS (version 22.0) and STATA-16.

## Results

### Demographic and occupational characteristics of the study participants

The details of the study participants’ demographic and occupational characteristics are described in Table 1. In this cross-sectional survey, of the 1,000 invited nurses from all over the country, 565 responded to the questionnaires, giving a 56.5% overall response rate. After removing the incomplete questionnaires, 547 nurses were recruited for this study, and among them, 235 (43.0%) were working in tertiary level healthcare facilities, 170 (31.1%) in secondary level healthcare facilities, and 142 (26.0%) in primary level healthcare facilities. Among the participants, 226 (41.3%) were frontline nurses, and 321 (58.7%) worked as second-line nurses. The median age of the participants was 26 (interquartile range 24–29) years. The majority of the respondents (66.0%) were female. In terms of educational level, a total of 253 (46.3%) respondents had diploma degrees, 184 (33.6%) had bachelor degrees, and 110 (20.1%) had master’s degrees in the related field. However, almost one-third of the respondents (31.6%) reported facing emotional abuse for being healthcare workers and working in this COVID-19 pandemic situation. Nearly half of the nurses (46.2%) did not get complete PPE from the authorities during their practice.

**Table 1:** Demographic and occupational characteristics of study participants (*n*=547)

Characteristic	***N*** (%)
**Median age (IQR), year =** 26 (24–29)	
**Gender**	
Male	186 (34.0)
Female	361 (66.0)
**Type of healthcare facility**	
Tertiary	235 (43.0)
Secondary	170 (31.1)
Primary	142 (26.0)
**Type of job**	
Government	302 (55.2)
Private	245 (44.8)
**Educational level**	
Diploma in nursing	253 (46.3)
B.Sc. in nursing	184 (33.6)
Masters	110 (20.1)
**Area of residence**	
Divisional city	346 (63.3)
District town	110 (20.1)
Sub-district	91 (16.6)
**Marital status**	
Unmarried	289 (52.8)
Married	258 (47.2)
**Professional title**	
Senior staff nurse	519 (94.9)
Nurse-in-charge	28 (5.1)
**Working position**	
Frontline	226 (41.3)
Second-line	321 (58.7)
**Had complete PPE during working**	
Yes	294 (53.8)
No	253 (46.2)
**Faced any emotional abuse**	
Yes	173 (31.6)
No	374 (68.4)

### Mental health symptoms and their measurements

For the mental health condition of the nurses amid the COVID-19 pandemic, as measured by DASS-21, we found depression in 276 (50.5%), anxiety in 283 (51.8%), and stress in 228 (41.7%) of the study participants (Table 2). The reported mean depression subscale score of DASS-21 was 10.89 (SD 9.49). One hundred and ninety-two (69.6%) of the nurses scored moderate to extremely severe depression from 276 nurses who scored positive for depression. The anxiety subscale mean value of DASS-21 was 9.54 (SD 8.87). Among the 283 participants screened positive for anxiety, 234 (82.7%) scored moderate to extremely severe. The mean score of DASS-21 stress subscale was 14.65 (SD 10.20). From 228 responders who screened positive for stress, 162 (71.1%) were found to be scored as moderate to extremely severe.

**Table 2:** Findings for different categories on the DASS-21 and IES-R during the COVID-19 outbreak in total cohort and their gender differences

Grades of symptoms	Overall, ***N*** (%)	Gender, ***N*** (%)			
		Male	Female	***χ***^**2**^	***p***-value
**Depression**					
Normal	271 (49.5)	102 (54.8)	169 (46.8)	10.258	
Mild	84 (15.4)	35 (18.8)	49 (13.6)		
Moderate	116 (21.2)	31 (16.7)	85 (23.5)		0.036*
Severe	35 (6.4)	9 (4.8)	26 (7.2)		
Extremely severe	41 (7.5)	9 (4.8)	32 (8.9)		
**Anxiety**					
Normal	264 (48.3)	115 (61.8)	149 (41.3)	29.503	
Mild	49 (9.0)	21 (11.3)	28 (7.8)		
Moderate	117 (21.4)	25 (13.4)	92 (25.5)		<0.001*
Severe	35 (6.4)	6 (3.2)	29 (8.0)		
Extremely severe	82 (15.0)	19 (10.2)	63 (17.5)		
**Stress**					
Normal	319 (58.3)	124 (66.7)	195 (54.0)	12.841	
Mild	66 (12.1)	23 (12.4)	43 (11.9)		
Moderate	75 (13.7)	21 (11.3)	54 (15.0)		0.012*
Severe	51 (9.3)	8 (4.3)	43 (11.9)		
Extremely severe	36 (6.6)	10 (5.4)	26 (7.2)		
**Impact of event**					
Normal	209 (38.2)	98 (52.7)	111 (30.7)	29.183	<0.001*
Mild	113 (20.7)	35 (18.8)	78 (21.6)		
Moderate	49 (9.0)	16 (8.6)	33 (9.1)		
Severe	176 (32.2)	37 (19.9)	139 (38.5)		

Chi-square test was applied to determine the difference of each distress between genders

*χ*^*2*^ chi-squared value

*Significant at *p* <0.05

Participants rated their impact of COVID-19 during the previous seven days of their interviews by IES-R scale. Among the participants, 338 (61.9%) of them was positive for psychological distress, and among them, 225 (66.6%) scored as moderate to severe level of distress (Table 2). The mean score for IES-R scale was 30.78 (SD 17.85); however, the mean scores for IES-R subsets for avoidance, intrusion, and hyperarousal were 1.25 (SD 0.88), 1.61 (SD 0.95), and 1.32 (SD 1.00) respectively.

In terms of different grades of psychological symptoms, there was a significant difference among male and female nurses (*p <0.05*). Psychological symptoms of depression, anxiety, stress, and impact of event were more prevalent in female nurses than male nurses (Table 2).

### Association between occupational factors and mental health symptoms

The univariate linear regression analysis revealed the association between the occupational factors and the mental health symptoms that are presented in Table 3. The nurses who worked in tertiary level healthcare facilities were significantly associated with higher scores in DASS-21 anxiety subscale (*p=0.041*) and DASS-21 stress subscale (*p=0.031*) compared with the nurses who worked in primary level healthcare facilities. On the other hand, the participants who completed diploma in nursing that is the lowest degree were significantly associated with lower scores in DASS-21 depression subscale (*p=0.030*), DASS-21 anxiety subscale (*p=0.015*), DASS-21 stress subscale (*p <0.001*), and IES-R scale (*p=0.039*) compared with those who completed master’s degrees that is the highest degree among the participant nurses. The nurses who reported of having complete PPE during working were significantly associated with lower scores in DASS-21 depression subscale (*p=0.001*), DASS-21 anxiety subscale (*p=0.005*), and DASS-21 stress subscale (*p <0.001*) compared with those who have no complete PPE during working. The nurses who faced any emotional abuse working in this pandemic situation were significantly associated with higher score in the DASS-21 depression subscale (*p <0.001*), DASS-21 anxiety subscale (*p <0.001*), DASS-21 stress subscale (*p <0.001*), and IES-R scale (*p <0.001*). Other occupational factors, including type of job, professional title, and working position, were not significantly associated with DASS-21 subscales and IES-R scores.

**Table 3:** Association between occupational factors and different mental health symptoms among nurses during the COVID-19 outbreak (univariate linear regression)

Variables	Depression		Anxiety		Stress		Impact of event	
	***B*** (95% CI)	***p-***value	***B*** (95% CI)	***p-***value	***B*** (95% CI)	***p-***value	***B*** (95% CI)	***p-***value
**Type of healthcare facility**								
Tertiary	1.10 (−0.87 to 3.08)	0.272	1.92 (0.08 to 3.76)	0.041*	2.33 (0.22 to 4.45)	0.031*	2.09 (−1.61 to 5.79)	0.267
Secondary	−1.37 (−3.48 to 0.74)	0.201	−0.82 (−2.79 to 1.15)	0.414	−0.72 (−2.98 to 1.54)	0.532	−3.59 (−7.55 to 0.37)	0.075
Primary	Reference		Reference		Reference		Reference	
**Type of job**								
Government	−1.15 (−2.75 to 0.45)	0.159	0.62 (−0.88 to 2.12)	0.416	0.66 (−1.06 to 2.39)	0.451	0.96 (−2.06 to 3.97)	0.534
Private	Reference		Reference		Reference		Reference	
**Educational level**								
Diploma in nursing	−2.35 (−4.46 to −0.23)	0.030*	−2.44 (−4.42 to −0.47)	0.015*	−4.27 (−6.52 to −2.02)	< 0.001*	−4.17 (−8.14 to −0.20)	0.039*
B.Sc. in nursing	0.15 (−2.08 to 2.38)	0.896	0.01 (−2.08 to 2.09)	0.999	0.57 (−2.94 to 1.81)	0.639	1.42 (−2.77 to 5.61)	0.505
Masters	Reference		Reference		Reference		Reference	
**Professional title**								
Senior staff nurse	0.32 (−3.28 to 3.96)	0.854	2.53 (−0.85 to 5.91)	0.142	−0.37 (−4.26 to 3.52)	0.853	−0.45 (−7.26 to 6.36)	0.896
Nurse-in-charge	Reference		Reference		Reference		Reference	
**Working position**								
In frontline	0.01 (−1.62 to 1.63)	0.997	0.82 (−0.69 to 2.34)	0.285	1.12 (−0.62 to 2.86)	0.205	1.30 (−1.74 to 4.35)	0.401
In second-line	Reference		Reference		Reference		Reference	
**Had complete PPE during working**								
Yes	−2.68 (−4.26 to −1.10)	0.001*	−2.15 (−3.63 to −0.66)	0.005*	−3.50 (−5.19 to −1.80)	< 0.001*	−2.68 (−5.68 to 0.32)	0.080
No	Reference		Reference		Reference		Reference	
**Faced any emotional abuse**								
Yes	5.51 (3.86 to 7.16)	< 0.001*	5.94 (4.42 to 7.47)	< 0.001*	6.67 (4.92 to 8.43)	< 0.001*	11.46 (8.38 to 14.54)	< 0.001*
No	Reference		Reference		Reference		Reference	

*B* unstandardized coefficient (the negative value means better mental health), *CI* confidence interval

*Significant at *p* <0.05

## Discussion

The current study investigated the overall mental health condition and the impact of COVID-19 on the mental health among the nurses of Bangladesh. To our knowledge, our study is the first of its kind during this COVID-19 pandemic that has been carried out among the nurse community to provide the spotlight on this neglected category of health professionals in Bangladesh.

This study revealed that 61.9% of nurses in our sample suffered from some degree of mental distress during the COVID-19 outbreak in Bangladesh; 50.5% was documented to have some degree of depression, 51.8% had some degree of anxiety, and 41.7% had some degree of stress. A similar study conducted in Nepal found high rates of psychological distress (41.9% had symptoms of anxiety, and 37.5% had symptoms of depression) during the COVID-19 outbreak among healthcare workers [17]. Another study conducted among the Chinese healthcare workers also reported symptoms of depression at 50.4%, anxiety at 44.6%, and distress at 71.5% [14]. A multinational study revealed low psychological impact (7.4%), depressive symptoms (10.6%), anxiety symptoms (15.7%), and stress level (5.2%) during this pandemic as compared to our study findings, conducted among the healthcare workers in Singapore and India [22]. This difference might be due to the variations in time of conducting the studies and also variations in demographic profiles of study participants. Health care workers exert a complicated psychological response to an epidemic of infectious diseases. Psychological distress may result from the thoughts of insecurity or lack of control and poor self-esteem, higher infection rate, well-being of the family and friends, workloads, and loneliness due to the quarantine [26, 27]. In addition, predictable stock shortages and a growing flow of reported and real COVID-19 reports lead to the stresses and worries among the healthcare staff [28].

In this study, between both genders, males had lesser depression, anxiety, and stress level and psychological effect during the COVID-19 outbreak as compared to their female counterparts. This finding was similar to the other studies where females suffered greater mental distress and poor mental health outcome during this COVID-19 outbreak [14, 23, 29].

The nurses of the tertiary level healthcare facilities are suffering from higher level of anxiety and stress as revealed in our study. In China, a multicenter study suggested that nurses of secondary hospitals experienced more depression and anxiety [14]. However, in Bangladesh, the nurses of tertiary level healthcare facilities had to deal with a huge number of patients compared to the primary or secondary level healthcare settings which might interact with the mental health during COVID-19 [30].

Our study showed that nurses who had a lower educational degree experienced lower impact from the COVID-19 pandemic psychologically and lower depression, anxiety, and stress scores, suggesting less mental distress than the nurses who had higher education. However, a study in the UK suggests that it was less stressful to work with patients if the nurses have higher education and strategies to manage mental health problems [31]. This contrary finding might be because, in healthcare settings in Bangladesh, less educated nurses had less responsibilities and leadership roles, so less exposure to potentially stressful situations.

Our study outcomes indicate that during the pandemic, the preventive arrangements taken to control the transmission of COVID-19 may have had protective psychological effects. The evidence from 2003 SARS-CoV epidemic study revealed that moderate anxiety had an association with the practice of high level of preventive measures [32]. Our outcome is also following this same trend. The safety measures for the nurses, particularly having complete PPE during working, come with low mental distress. Another significant finding in our study from regression analysis is that the nurses who faced any emotional abuse for being healthcare workers and working in COVID-19 pandemic situations were associated with higher levels of depression, anxiety, stress, and psychological impact. Similar study conducted in Nepal found that healthcare workers who faced stigma during the COVID-19 outbreak were more at risk of developing mental health outcomes [17]. In Bangladesh, health care workers are facing social stigma since the outbreak of COVID-19 [33]. A research from China also found that individuals with a greater propensity to communicate their mental health distress is the product of social stigma [34].

In this study, no significant association has been established between psychological outcomes and the working position of the nurses during COVID-19. This study finding is similar to the results of studies conducted in Nepal and Italy [17, 35]. However, several other studies indicate that nurses who are involved with managing COVID-19 patients directly were at the highest risk of getting the diseases [36, 37], and they experienced more adverse psychological outcomes than their counterparts [14, 38]. As most of the COVID-19 cases had mild symptoms, working position might not have contributed to a significant difference in mental health outcomes. The similarity between the frontline and second-line nurses regarding psychological outcomes in our study may be due to that second-line nurses also remain suspicious and have fear of getting affected as they deal with the patients whom they do not know are infected or not. Further research might be required in this area to confirm this finding as the association might vary over the course of the epidemic in the country.

Therefore, to improve the mental health and well-being of the nurses, multi-disciplinary interventions are necessary by addressing psychological outcomes. Dedicated counseling should be arranged to support the psychological well-being of nurses to help improve their morale. The government and the health authorities should ensure that there are adequate supplies of protective equipment for the nurses during working in the pandemic. The government should also take initiatives to prevent social stigma and uphold the position of healthcare workers as frontline fighters against COVID-19 so that they might not face any emotional abuse for being a healthcare worker when they are working amid COVID-19 pandemic. Since these results pertain to the duration of the pandemic in Bangladesh, broader longitudinal research should be undertaken in the current time to direct policymakers in recognizing the psychological effects of COVID-19.

### Limitations

There are some limitations in this study. First, the intrinsic nature of the research is confined to nurses with internet access, as is the sampling technique. Second, the causal explanation was not possible due to the cross-sectional design of the research. Third, this survey screened for symptoms and not a detailed mental health assessment that confirms the diagnosis, so it did not take into account nurses who might have pre-existing mental health issues or who might develop symptoms due to factors other than COVID-19. Fourth, in this study, just under half of the potential respondents did not participate, so there might be more motives for those with symptoms to respond and might over-represent the prevalence of symptoms in the nursing population.

Regardless of the constraints, this study indicates the primary fundamental information on the real degree of psychological symptoms among Bangladeshi nurses and how the mental well-being of nurses is varied during this pandemic.

## Conclusions

A high prevalence of depression, anxiety, stress, and psychological impact was observed in the Bangladeshi nurses working in hospitals during the COVID-19 outbreak. Female nurses were more prone to higher psychological impact and adverse mental health outcomes compared to male nurses. The factors that predicted higher impact and adverse mental health are not having complete PPE during working and facing any emotional abuse by the nurses during COVID-19. For healthcare professionals, during a pandemic, timely psychological support and intervention are required. Protecting health care workers by ensuring protective equipment and upholding their position by preventing social stigma are important components of public health interventions to counter the COVID-19 pandemic. It is essential to urgently implement specific measures to promote mental health well-being in nurses, especially for females exposed to COVID-19.

## Data Availability

The datasets used and/or analyzed during the current study are available from the corresponding author on reasonable request.

## Abbreviations

COVID-19: Coronavirus disease-19
DASS-21: Depression, Anxiety and Stress Scale-21
IES-R: Impact of Event Scale-Revised
PPE: Personal protective equipment
